# T-Cell B-Rich Lymphoma Presenting as Renal Colic with Positivity of CD3

**Published:** 2019-01-01

**Authors:** Hasan Nabil Al Houri, Tagrid Younes Ahmad, Sarah Zaher Adden, Wisam Hikmat Assad, Ammar Raiy

**Affiliations:** 1 Department of Internal Medicine, Al Assad University Hospital and Al Mouwasat University Hospital, Damascus, Syria; 2 Department of Internal Medicine, Tishreen Hospital, Damascus, Syria; 3 Department of Ophthalmology, Al Mouassat University Hospital, Damascus University, Damascus, Syria; 4 Department of Pathology, Al Mouassat University Hospital and Syrian Private University, Damascus, Syria; 5 Department of Kidney Transplant, Al Mouassat University Hospital, Damascus, Syria

**Keywords:** T-cell-rich B-cell lymphoma; B-cell lymphoma; TCRBCL; Renal colic

## Abstract

T-Cell Rich B-Cell Lymphoma (TCRBCL) is relatively a new entity, lately classified as a morphologic variant of Diffuse Large B-cell lymphomas (DLBCL). It consists (1-3) % of all B-cell lymphomas. The rate is far less when describing cases of primary splenic involvement with TCRBCL. Pathologically, TCRBCL is described as a limited number of scattered, large, atypical b-cells embedded in a background of abundant t-cells and frequently histiocytes. The similarity of this malignancy with other types makes it difficult to distinguish between them. Thus, it needs expertise in both clinical and pathological fields to make the right diagnosis.Here, we present a case of an adult male patient whose first presentation and previous medical history of renal colic misguided the initial diagnosis and suggested another colic episode as the underlying ailment. However, further physical, radiological and histopathological investigations uncovered the presence of primary TCRBCL within spleen with no involvement of other sites. Moreover, unusual pathologic finding of CD3 positivity was proved by immunohistochemistry.

## Introduction

 T-Cell Rich B-Cell Lymphoma (TCRBCL) was first described in 1988^1^. In 2001, WHO stated it as a distinct entity^2^. However, since 2008, it has been classified as a morphologic variant of Diffuse Large B-Cell Lymphoma (DLBCL)^3^, comprising 1-3% of all B-cell lymphomas^4^. Moreover, the rate is far less when mentioning primary splenic involvement with TCRBCL^5^. This subtype is characterized by an aggressive clinical behavior, and often presents with advanced stage, splenomegaly and bone marrow involvement^4^. Clinical presentations vary from isolated splenomegaly to non-specific systemic illness symptom. Pathologically, TCRBCL is described as limited number of scattered, large, atypical b-cells embedded in a background of abundant t-cells and frequently histiocytes^6^.

Here, we present a case of 43-year-old patient, which firstly was misdiagnosed as renal colic, and then accidently found to have Primary TCRBCL in spleen with no involvement of other sites. Moreover, unusual pathologic finding of CD3 positivity was proved by immunhistochemistry.

## Case presentation

 A 43-year-old male patient, with previous history of renal colic, presented to Al-Mowasat University Hospital in 2016 with a chief complaint of colic pain in the left upper quadrant. Physical examination was within normal limits. On ultrasonography (US), the spleen measured 14cm in its greatest dimension. Besides, it showed a hypo-echoic cyst-like mass that measured about 7cm. The patient underwent diagnostic/therapeutic splenectomy. During surgery, there were some adhesions between the spleen and the diaphragm. Afterwards, the resected spleen was sent to the pathology department of the hospital. Macroscopically, the specimen measured (14×10×5) cm, and weighed 355g. On resecting, a gross tumor of (7×6×5.5) cm was noted within the spleen invading the capsule. Histosections were consistent with large B-cell lymphoma. Immunohistological staining was performed and showed the following: large cells were positive for CD20, BCL-2, whereas the surrounding small cells were stained positive for CD3. Based on pathological findings, a diagnosis of T-cell-rich B-cell lymphoma was established. Further staging including peripheral blood smear, bone marrow biopsy and CT scan for neck, chest and abdomen became negative. Thus, the involvement of other sites was ruled out. Later, the patient completed immunochemotherapy courses of R-CHOP. At the time of the follow-up examination 3 years after the initial diagnosis, the patient was alive, well and without evidence of recurrence.

**Histopathology sections**

**Figure 1:**
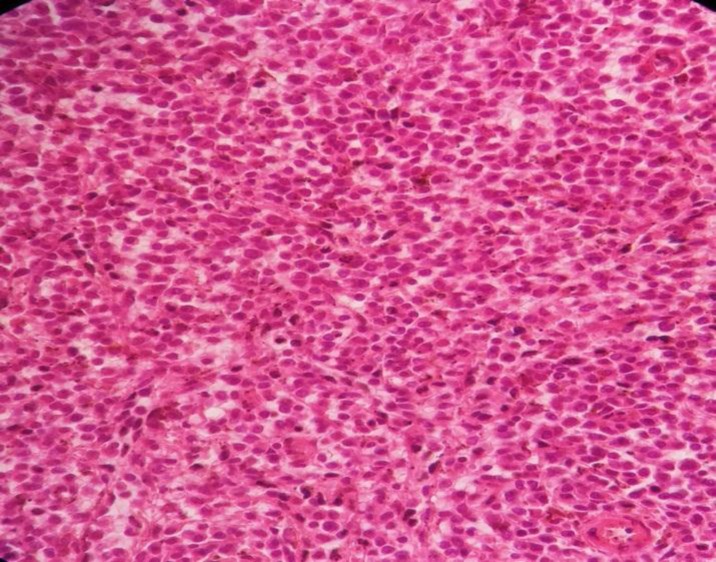
Spleen tissue biopsy, infiltration of red pulp cords, sinusoids and scattered residual white pulp islands by sheets of pleomorphic large cells resembling popcorn cells

The sections revealed splenic tissue with infiltration of red pulp cords, sinusoids and scattered residual white pulp islands by sheets of pleomorphic large cells resembling popcorn cells. These cells had pale and indistinct cytoplasm, vesicular nuclei with small central nucleoli and frequent mitotic figures. There was a background of small lymphocytes and often histiocytes. The tumor cells invaded the spleen capsule. Neither granuloma nor necrosis was observed (Figure 1).

**Figure 2:**
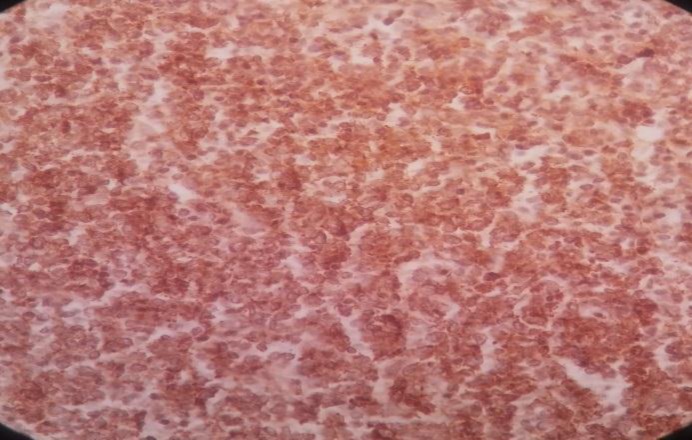
Spleen tissue biopsy, positive for CD20

**Figure 3:**
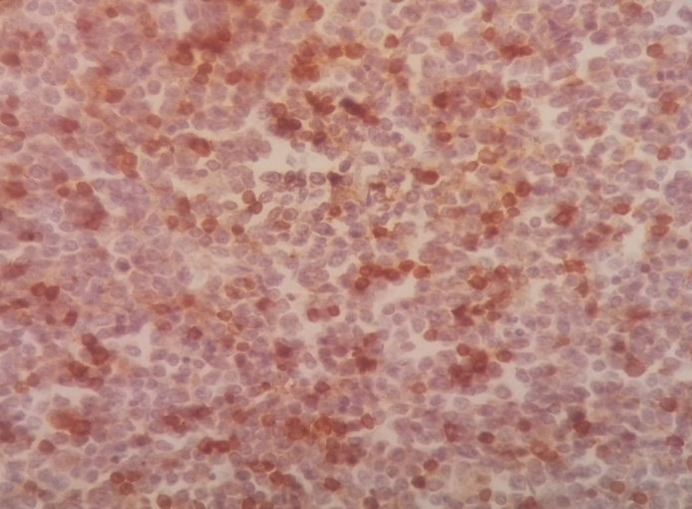
Spleen tissue biopsy, positive for CD3

**Figure 4:**
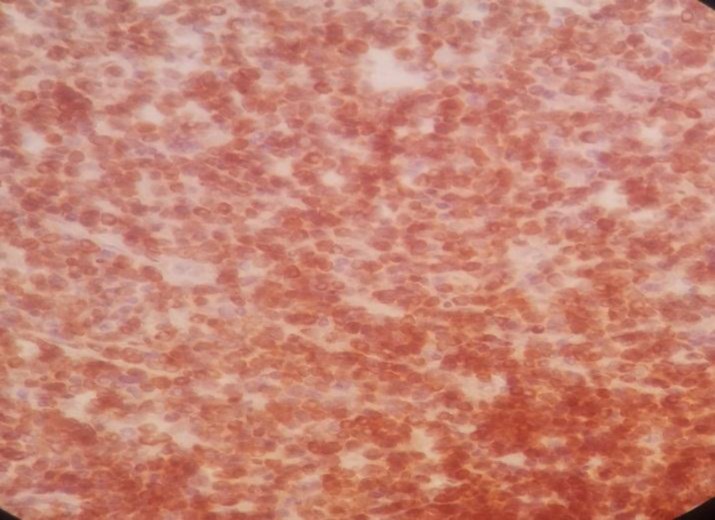
Spleen tissue biopsy, positive for BCL-2

**Figure 5:**
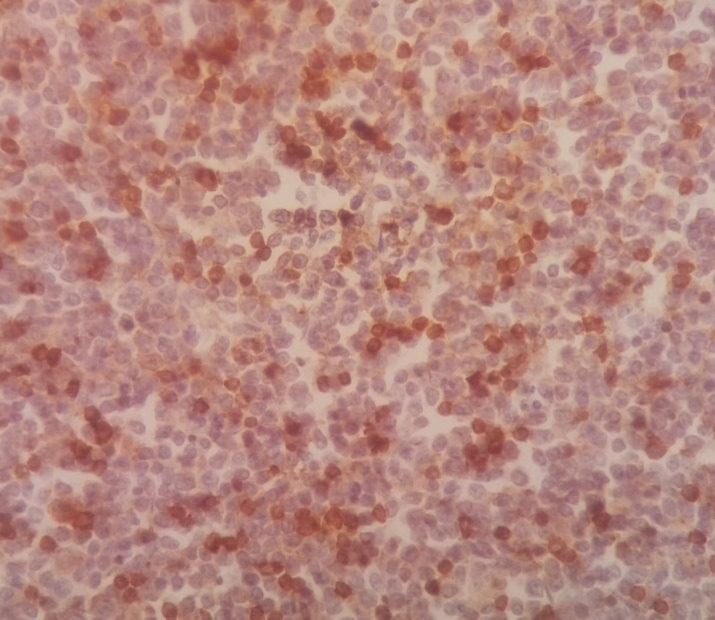
Spleen tissue biopsy, positive for CD30

**Figure 6:**
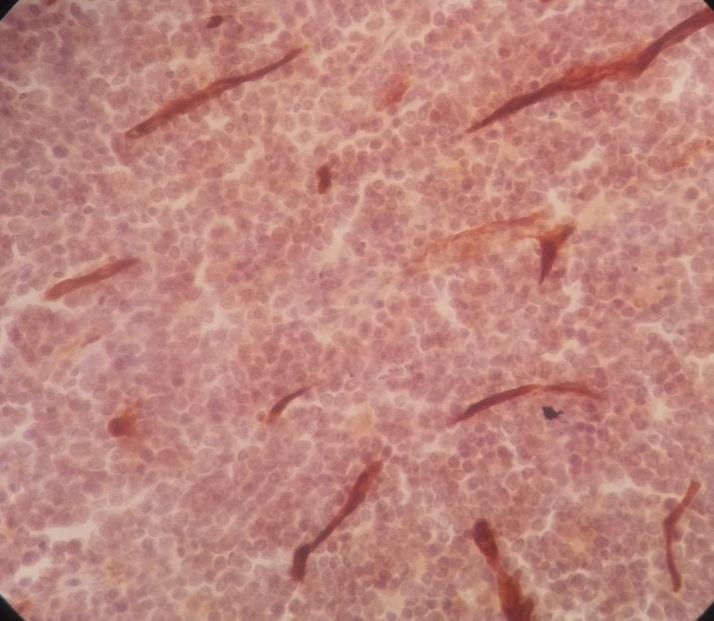
Spleen tissue biopsy, positive for CD34

**Figure 7:**
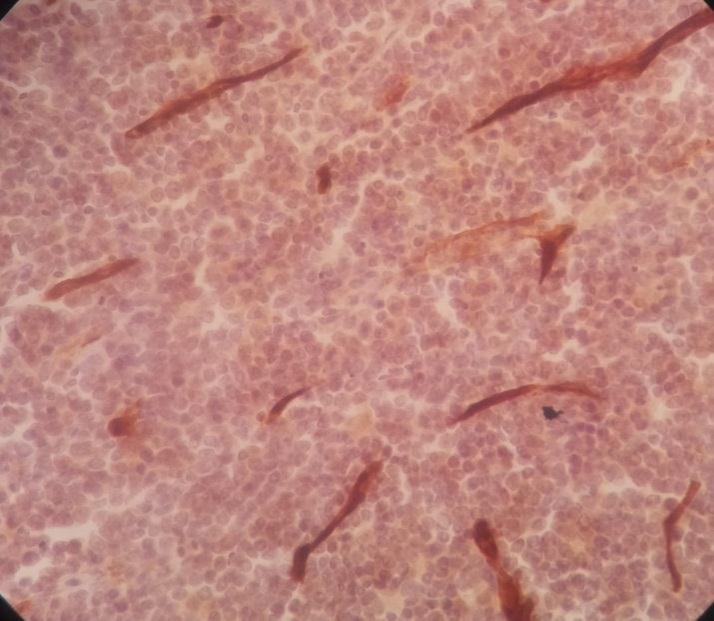
Spleen tissue biopsy, negative for CD15

**Figure 8:**
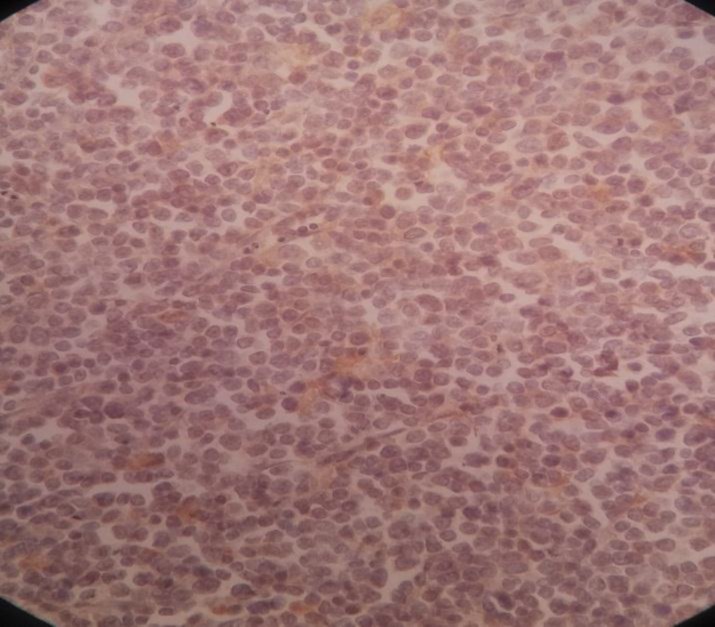
Spleen tissue biopsy, negative for EMA. Bone marrow Biopsy revealed thin anastomosing bony trabeculae separating 15 hematopoietic spaces with normal cellularity (60%)(3/5)(Figure 9)

Immunohistochemistry staining was positive for CD20 on large cells, CD3 on small lymphocytes, BCl2 on large cells, CD30 on some large cells, and CD34 on residual red pulp sinusoids, but negative for both CD15 and EMA (Figures 2-8).

Bone marrow Biopsy

**Figure 9:**
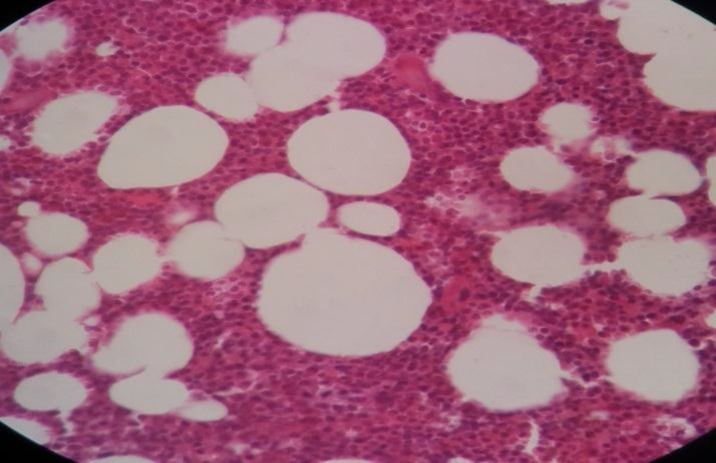
Bone marrow Biopsy

 The erythroid, myeloid, megakaryocytic lineages were well differentiated (M/E =5/2). No sign of infiltration, fibrosis, and proliferative disease was observed within BM.

The immunohistochemistry staining revealed positivity for MPO, LCA, CD20, and CD3 within normal distribution (Figures 1012)

**Figure 10:**
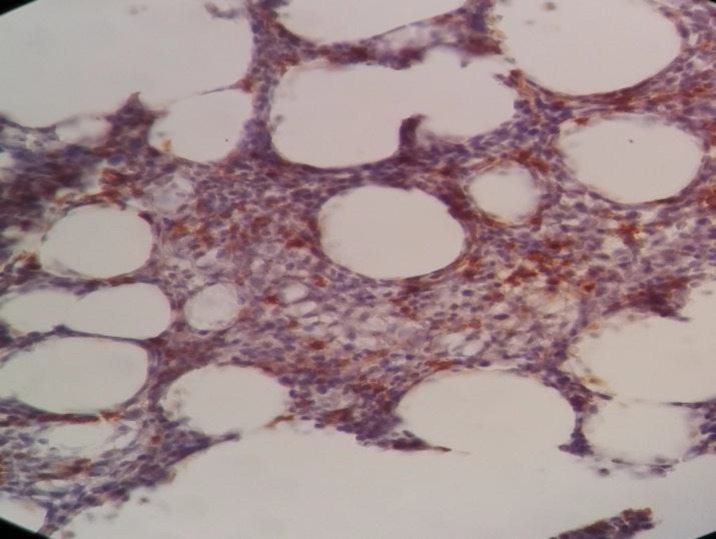
Bone marrow Biopsy, LCA within normal distribution

**Figure 11:**
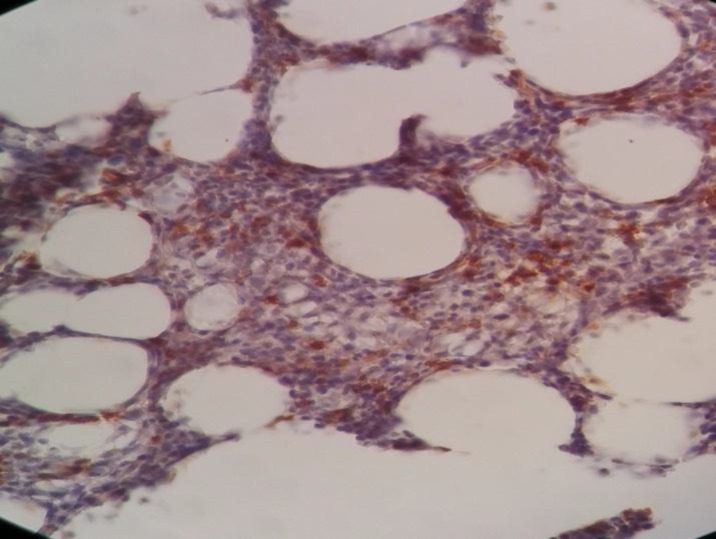
Bone marrow Biopsy, CD20 within normal distribution

**Figure 12:**
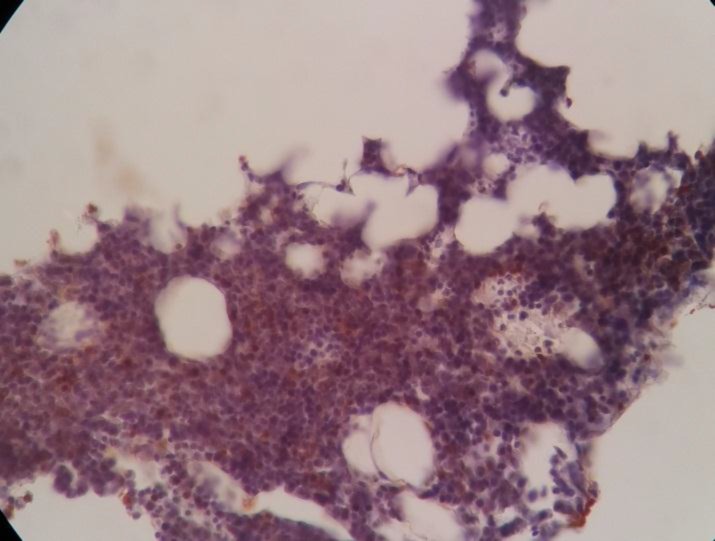
Bone marrow Biopsy, CD3 within normal distribution

The histopathological sections confirmed the diagnosis of TCRBCL of the spleen.

## Discussion

 Primary splenic lymphomas are extremely rare in comparison with secondary ones, and account for less than 1% of all lymphomas^7^. Non-Hodgkin lymphoma of B-cell origin is the most common type of primary splenic lymphoma^8^, while the occurrence of T-cell/histiocyte-rich large B-cell lymphoma (T/HRLBCL) is scarce^8^. TRLBCL was first described in 1988 by Ramsay^1^. Then, the term was expanded by Delabie in 1992 who reported six cases of T/HRLBCL^9^. In 2001, the World Health Organization (WHO) considered it as a distinct entity of aggressive lymphoma^2^.  In 2008, this subtype was classified as a variant of Diffuse Large B-Cell Lymphoma (DLBCL), forming less than 10% of DLBCL^3^. T/HRLBCL has a male predilection, and usually presents in the fourth decade of life, compared with DLBCL that occurs equally in both genders and usually in the sixth decade of life^10^^,^^11^. T/HRLBCL has higher frequency than DLBCL to infiltrate the spleen, liver and bone marrow^12^. Furthermore, they are more likely to develop B symptoms, drenching night sweats, weight loss >10% and fevers^10^^,^^11^^,^^13^. In this case, the patient presented with colicky pain in the left upper quadrant which was misdiagnosed as renal colic, but further investigations revealed a cyst-like mass in the spleen. Then, the patient underwent diagnostic and therapeutic splenectomy. The biopsy revealed the presence of TCRBCL as the primary malignancy. Our medical literature search discovered no report of patients with the same presentations. 

T/HCRLBCL shares several morphological and immunophenotypic similarities with classical Hodgkin lymphoma (CHL), nodular lymphocyte-predominant Hodgkin lymphoma (NLPHL), and peripheral T-cell lymphoma, thus these are sometimes referred to as "grey zone lymphomas"^14^. It is difficult to distinguish T/HRLBCL from such similar neoplasms. Therefore, the diagnosis of T/HRLBCL requires experienced pathologist and reliable immunohistochemical (IHC) analysis^12^. Histologically, it is composed of few (usually <10%) large B cells that are disseminated in the background of abundant T-lymphocytes with or without histiocytes^3^. T/HRLBCL demonstrated morphologic and immunophenotypic positivity for CD20 and CD45 (characteristic for malignant B- lymphocytes), Bcl-6 and CD3 (characteristic for the polyclonal T lymphocytes that form the prominent component of T/HRLBCL) with or without the presence of CD68 (characteristic for histiocytes) ^4^^,^^12^ , but showed variable positivity for Bcl-2 and Epithelial Membrane Antigen (EMA). It is noteworthy that T/HRLBCL was negative for CD15 and rarely positive for CD30 ^4^^,^^12^. In this case, the specimen was positive for CD20, CD45 and CD3 and some cells stained for CD30, which was considered to be a rare finding in T/HRBCL.

## CONCLUSION

Unusual symptom with systemic examination led to early detection of a high-grade malignancy. Primary splenic T/HRLBCL is an uncommon hematologic neoplasm. However, its diagnosis forms a major dilemma for pathologists since it is difficult to distinguish T/HRLBCL from other similar neoplasms such as (CHL) and (NLPHL). The integration between the morphologic features and immunohistochemistry analysis is fundamental to diagnose primary splenic T-cell/ histiocyte- rich large B-cell lymphoma.
